# Using Gamification and Social Incentives to Increase Physical Activity and Related Social Cognition among Undergraduate Students in Shanghai, China

**DOI:** 10.3390/ijerph16050858

**Published:** 2019-03-08

**Authors:** Dandan Mo, Mi Xiang, Mengyun Luo, Yuanyuan Dong, Yue Fang, Shunxing Zhang, Zhiruo Zhang, Huigang Liang

**Affiliations:** 1 School of Public Health, Shanghai Jiao Tong University, Shanghai 200025, China; modandan1314@126.com (D.M.); kou_mitsu@hotmail.com (M.X.); my19930220@126.com (M.L.); dyy714@foxmail.com (Y.D.); fangyue@sjtu.edu.cn (Y.F.); 2 School of Public Health, and Charles Perkins Centre, University of Sydney, Sydney, NSW 2006, Australia; 3 Child Health Advocacy Institute, Shanghai Children’s Medical Center, School of Medicine, Shanghai Jiao Tong University, Shanghai 200127, China; 4 Scarsdale High School, Scarsdale, NY 10583, USA; szhang20@scarsdaleschools.org; 5 College of Business, East Carolina University, Greenville, NC 27858, USA

**Keywords:** physical activity, gamification, social incentives, social networking service, WeChat, undergraduate students

## Abstract

Gamification and social incentives are promising strategies to increase the effectiveness of web-based physical activity (PA) interventions by improving engagement. In this study, we designed a PA intervention integrating gamification and social incentives based on the most popular social networking service in China, WeChat. A controlled trial involving 52 Chinese undergraduate students was implemented to evaluate the effectiveness of the intervention. Subjects in the intervention group received a 7-week intervention. PA behavior and related social cognitive variables according to the theory of planned behavior were measured at the baseline and after the intervention. Daily physical activity duration was measured during the intervention. The results showed that PA-related subjective norms, perceived behavior control, and intention, as well as self-reported vigorous physical activity and moderate physical activity in the intervention group, were increased after the intervention, compared with the control group (*p* <0.05). During the intervention, perceived daily physical activity duration in the intervention group was on the rise, while it declined in the control group (*p* <0.001). The findings indicate that WeChat-based intervention integrating gamification and social incentives could effectively increase subjectively measured PA and related social cognition among Chinese undergraduate students and that it is a promising way to ameliorate the problem of insufficient PA among youths.

## 1. Introduction

Adequate and regular physical activity (PA) is widely accepted as a way to reduce all-cause mortality and to promote considerable health outcomes [1]. The benefits of PA include reduced risk and burden of many noncommunicable diseases (such as cardiovascular diseases, certain kinds of cancers, and diabetes), increased life expectancy, and improved quality of life [2,3,4]. The World Health Organization (WHO) has recommended that adults aged 18–64 years do at least 150 min of moderate-intensity PA or 75 min of vigorous-intensity PA or an equivalent combination of moderate- and vigorous-intensity PA throughout the week [5]. If this guideline of PA could be followed and become routine at an early age, health benefits could be experienced throughout one’s lifespan. Unfortunately, previous studies have shown that PA declines by an estimated 7% per year and sedentary behavior increases with age throughout adolescence [6,7]. Among those at the ages of 18–22, a high prevalence of physical inactivity was found during the period of attendance at universities or colleges. According to the recommendation for PA by the WHO, Keating XD et al. suggested that 30–50% of undergraduate students are inactive [8]. Pengpid et al. reported that about 41.4% of undergraduate students in 23 countries do not get enough PA [9]. The situation is even worse in China. The “2010 National Physical Fitness and Health Surveillance” reported that 77.3% of Chinese adolescents failed to meet the recommended levels of PA [10]. Wu et al. found that 68.8% of Chinese undergraduate students are physically inactive in a large-scale research [11], and a recent study indicated that this rate is still as high as 60.14% [12]. Thus, it is imperative to increase PA among undergraduate students in China. Moreover, Chinese undergraduates usually live on campus. With the abatement of influences from family, they are inclined to develop unhealthy behaviors and lifestyles. The college period, therefore, is a critical period to intervene with students to increase their PA to obtain lifelong benefits.

To explain why youths undertake PA, the theory of planned behavior (TPB) has been widely applied and validated in many countries, including the US [13,14], the UK [15], Australia [16], and China [17]. According to the TPB, the best predictor of a behavior is the intention to perform the behavior. Intention is determined by attitude toward the behavior, subjective norms, and perceived behavioral control. Attitude is the overall feeling of liking or disliking toward the behavior. Subjective norms are the motivations or pressures that one can perceive from his or her social contacts. Perceived behavioral control is one’s belief about the presence of factors that may make it easier or harder to perform the behavior, and it reflects one’s beliefs about whether he or she can perform the behavior (self-efficacy) [18]. In this framework, aspects of attitude, subjective norms, and perceived behavior control should be taken into consideration to promote one’s intention when developing strategies for behavior change. Previous interventions based on TPB have shown benign effects on increasing PA [19,20]. However, although TPB has provided a theoretical framework, some interventions have achieved few effects due to lack of practical techniques, and the traditional supervised PA intervention has usually suffered from high costs but relatively low popularity, low convenience, and low flexibility, as well as low anonymity [21]. Fortunately, the advancement of internet technologies has bought forth revolutionary opportunities in PA interventions.

The rapidly expanding availability of smartphones and mobile networks contributes to the high popularity of social networking services (SNS), such as Facebook and WeChat, which provide an appropriate platform for real-time communication and information sharing, as well as participating in a variety of virtual activities. With such broad accessibility, mobile SNS tools are perceived as convenient by the users, given their flexibility, anonymity, and low cost [21,22,23]. However, not all interventions delivered by SNS have achieved significant effects, and researchers have realized that intervention effects will be limited if subjects have a low level of adherence and are not adequately engaged [24]. Therefore, there is a strong need to ameliorate PA intervention design to provide incentives for participants to persist in PA [25]. Recently, the application of gamification and social incentives have shed light on addressing this challenge.

Gamification, the application of game design elements, such as points and levels in nongame conditions, is being increasingly utilized to promote changes in health behaviors, especially PA [26,27,28]. Previous studies indicated that gamification, if used properly, could increase adherence to and the effectiveness of PA interventions [29,30,31]. This is because designs based on gamification could satisfy intrinsic psychological needs [32], make the process interesting, enrich user experience, improve engagement, and stimulate enduring involvement [24]. Social incentives refer to the influences that motivate individuals to adjust their behaviors based on social contacts [33]. Typical modes of social incentives, such as collaboration, accountability, competition, and peer support, could be felicitously leveraged within gamification interventions and thus provide a practical approach to increase engagement in PA intervention [34,35].

So far, little research has been done to combine TPB with gamification and social incentives to enhance long-term motivation to increase PA. As some researchers have lamented, most previous gamification applications have not appropriately leveraged principles from theories of health behavior [29,36,37]. To probe into the applicability and effectiveness of gamification and social incentives in TPB-based intervention, we developed a PA intervention for undergraduates, based on WeChat. WeChat is the Chinese counterpart of Facebook and is the most popular SNS in China. According to the statistics reported by Tencent, the company which developed WeChat, there were 1.058 million users active on WeChat in June 2018 [38]. Using WeChat has already become a daily routine for many Chinese people, especially youths. This success has benefited from the rich and convenient functions of WeChat. Users can send texts, voices, photos, videos, and files, as well as web links, and make voice calls or video calls to other users on WeChat. These functions make it easy to implement gamification and social incentives designs.

The main purpose of this study was to test the effectiveness of our PA intervention through a controlled trial by investigating whether it could improve subjectively measured PA and related social cognitive constructs based on TPB. Our hypothesis was that subjectively measured PA and related TPB constructs of the intervention group would increase after the intervention, compared with the control group.

## 2. Materials and Methods

### 2.1. Study Design

This study received ethical approval from the Ethics Committee of Shanghai Jiao Tong University. A non-randomized controlled trial involving one intervention group and one control group was conducted from 13 March 2018 to 1 May 2018 at Shanghai Jiao Tong University, consisting of a 1-week run-in period and a 7-week intervention period. The data were collected twice: at the baseline (T0, *n* = 54) and after the intervention (T1, *n* = 52). A total of 2 subjects (3.7%) dropped out during the study.

The participants were assigned to an intervention group or a control group. A WeChat group was created for each group by the investigators. The WeChat group for the intervention group and control group were called BIG INTERVENTION GROUP and BIG CONTROL GROUP, respectively. Educational materials about PA were posted in each WeChat group every Friday. The subjects in the intervention group received the intervention integrating gamification and social incentives, while those in the control group were only required to report their daily PA duration (DPAD) every day in the BIG CONTROL GROUP.

### 2.2. Recruitment and Participants

The participants were recruited from 18 February 2018 to 2 March 2018 at Shanghai Jiao Tong University by the posting of advertisements on notice boards and through official WeChat links and emails of the university. The participants interested in this study could get in touch with the investigators through the WeChat ID posted on the advertisement. Then participants were screened for eligibility via a questionnaire on WeChat. The eligibility criteria included the following: aged 18–24 years, owning a smartphone, and having an active WeChat account. The participants were excluded if they were already participating in a physical activity study, would participate in other physical activity programs during the study, responded “yes” to any questions on the physical activity readiness questionnaire indicating health risks for participating in physical activity, had been told not to exercise by a physician, were currently pregnant, or if there was any other concern that participation was unsafe or infeasible. Finally, 54 students enrolled in this study. The eligible participants completed informed consent procedures, which had been reviewed and approved by the university’s research ethics board.

### 2.3. Power Analysis

We conducted power analysis by using G*Power. To detect a medium effect (f = 0.25) in a repeated measures ANOVA (with 7 waves of measures) with 90% power at an alpha level of 0.05, a sample size of 22 participants (11 per group) was needed. With 52 participants (17 in intervention and 35 in control), we had sufficient power to detect the effect of the intervention.

### 2.4. Baseline Measures and Goal Setting

Before allocation, the investigators gave instructions to the participants through WeChat regarding how to record their total time of PA, including moderate-intensity and vigorous-intensity PA, and how to send it to the investigators every day in the roll-in week. Their baseline daily physical activity duration (DPAD) was calculated as the 7-day average.

After allocation but before the intervention started, each participant was informed of his or her baseline DPAD and was asked to set his or her goal of DPAD according to the gap between the total PA duration in the roll-in week and the recommended PA level by the WHO [5] and then send his or her goals to the investigators.

The recommended standard for the goal setting is shown in Table 1.

All the interactions between the investigators and participants were through WeChat except for one face-to-face kick-off meeting organized for the intervention group.

### 2.5. Intervention

We used WeChat as the social media platform to carry out the intervention. The intervention took place in WeChat groups. A WeChat group called BIG INTERVENTION GROUP for all the subjects in the intervention group was established by the investigators, and they were assigned into peer-support teams involving 5–6 members. Then, a WeChat group for each peer-support team was created by the investigators.

Before the intervention started, a kick-off meeting was organized by the investigators. All the subjects in the intervention group were required to attend in the flesh. The aims of the meeting were to ensure that the subjects in the intervention group fully understood what they needed to do and to foster familiarity and cohesion among their teammates, as well as elect their team leader, given that familiarity and friendship could increase effective and positive interaction in the social groups and thus promote engagement, as well as motivation [39]. They also signed a commitment pledge during the meeting to try their best to achieve their daily physical activity duration (DPAD) goal, because pre-commitment has been found to motivate behavior change [40,41]. Then, the intervention group was exposed to the intervention for 7 weeks.

#### 2.5.1. Designs to Enhance Social Incentives

Evidence has shown that sharing information about the target behavior in the intervention with social contact and acting in groups rather than individually could raise subjects’ involvement with the intervention [34]. Peer support, accountability, competition, and reward have also been proven to enhance social incentives [35]. Thus, we embedded these features into the intervention to stimulate enduring engagement:

1. Daily Report within Teams: The subjects were required to report their DPAD of the prior day and whether they had met their goals in their team WeChat group before 15:00 every day.
2. Peer Support: Praising teammates who achieved their DPAD goals and encouraging those who did not within their team WeChat group every day were also required. Criticism and ridicule, which might cause negative reactions, were not allowed.
3. Accountability: The leader of each team was endowed with the responsibility to remind their teammates and send peer support (PS) in the BIG INTERVENTION GROUP once all the teammates had reported and interacted before 15:00 every day.
4. Team Punishment: The team that sent PS later than 15:00 the most times during the week would receive a punishment during the weekend.
5. Team discussion: Educational materials about PA were posted by the investigators in each WeChat group on every Friday. The participants were guided to read it and discuss their gains, as well as the advantages and barriers of promoting PA within groups.
6. Competition and Reward: A team competition was held to stimulate the subjects to engage in teamwork, and all the subjects acted as raters to ensure participation. The team that got the highest score was rewarded with a virtual certificate.

#### 2.5.2. Designs to Enhance Gamification 

Points, ranking, punishments, and rewards are typical modes of gamification and have been confirmed as effective techniques to improve the interest, incentive, and purposefulness of non-game programs [27,32]. Several studies have indicated their potential in stimulating engagement in PA behavior change, especially for youths [31,42]. Thus, the intervention was enriched by including gamification features as follows.

1. Points: Every team was endowed with 100 points for 1 week. Each day, if they failed to send PS before 15:00, 10 points were deducted. If any member was absent in the team discussion, 30 points were deducted. This design was based on the following three psychological principles: individuals tend to be more motivated by losses than gains [43], behavior is often better sustained by variable than by constant reinforcement [44], and individuals tend to be more motivated for aspirational behavior around temporal landmarks, such as the beginning of the week (the fresh start effect) [45].
2. Ranking: The ranking by the final points of each team for every week was announced in the BIG INTERVENTION GROUP on every Sunday.
3. Punishment: The team at the bottom of the ranking was required to perform a talent show in the BIG INTERVENTION GROUP (posting a voice message of a song or standup comedy or a video of a dance performance were all acceptable). Utilizing such performance as the mode of punishment could urge participants to follow the rules and avoid embarrassment, and it could also lighten up the atmosphere, thus improving compliance.
4. Rewards: At the end of the intervention, each member of the team that accumulated the highest points was rewarded with a diploma and a small prize, such as a mug or a notebook.

### 2.6. Outcome Measures

#### 2.6.1. Physical Activity (PA)

Self-administered short forms of the International Physical Activity Questionnaire (IPAQ) (for 15–69 years) were used to measure PA during the last 7 days. It contained 7 items regarding vigorous physical activities (VPA), moderate physical activities (MPA), walking, and sitting [46].

#### 2.6.2. Daily Physical Activity Duration (DPAD)

The DPAD was recorded on a spreadsheet embedded in WeChat. The participants input their DPAD on the spreadsheet and then posted it to their WeChat groups, so that their PA change over time was monitored by themselves and their teammates. The weekly PA duration for each week was also calculated.

#### 2.6.3. Theory of Planned Behavior Constructs

The questionnaire developed by Ajzen [47] was utilized to measure the four cognitive TPB constructs: attitude, subjective norms, perceived behavioral control, and behavioral intention. The participants’ own DPAD goal was defined as the target behavior.

Attitude was measured with 3 items (Cronbach alpha = 0.872), on a 7-point bipolar adjective scale, the choices were prefaced with the statement ‘‘For me, my DPAD goal would be…” followed by 3 items (bad–good, unpleasant–pleasant, and worthless–valuable), scored from 1 to 7.

7. Subjective norms were measured with 3 items (Cronbach alpha = 0.834), scored from 1 (extremely disagree) to 7 (extremely agree): (a) ‘‘Most people who are important to me approve of my DPAD goal”; (b). “Most people like me will meet my DPAD goal”; and (c). “Most people who are important to me think that I should try to meet my DPAD goal”.
8. Perceived behavior control was measured with 3 items (Cronbach alpha = 0.912), scored from 1 (extremely disagree) to 7 (extremely agree): (a) “I am confident that I can meet my DPAD Goal”; (b). “Whether or not I meet my DPAD Goal” is up to me; and (c). “I have the ability to meet my DPAD Goal”.
9. Intention was measured with 3 items (Cronbach alpha = 0.941), scored from 1 (extremely disagree) to 7 (extremely agree): (a) “I intend to meet my DPAD Goal”; (b). “I will make an effort to meet my DPAD Goal”; and (c). “I plan to meet my DPAD Goal”.

The subjects were required to complete the IPAQ and TPB questionnaires through an electronic link sent by the investigators on WeChat privately. They were not aware of each other’s responses.

### 2.7. Statistical Analysis

The data analysis was conducted with SPSS version 22. Missing values of demographics, TPB constructs, and physical activity measured by the questionnaire were deleted listwise, while missing values of DAPD were filled using multiple imputation in SPSS. Of the 2548 data of DPAD from the 52 participants during the 49-day measures, there were 5 missing values (about 0.20%) from 1 subject in the intervention group and 2 subjects in the control group. A total of 5 imputations were conducted using the following predictors of missing data: baseline DPAD, study group, calendar month fixed effects, week in the study, and a binary variable indicating the weekday or weekend. The imputed results were consolidated into 1 result by calculating the mean, using standard rules by Rubin [48]. Quantitative variables including age, body mass index (BMI), TPB constructs, PA, and DPAD were tested for normality and homogeneity of variance, and descriptive analyses for all the variables were conducted. The effect of the intervention on PA and related TPB constructs was tested using independent samples Mann–Whitney U test. Trends of DPAD for each week in the 2 groups were analyzed through repeated measures ANOVA. Prediction of the TPB constructs on PA was confirmed through ordinary least squares regression analyses with path analysis. The statistical significance was set at *p* <0.05.

## 3. Results

### 3.1. Baseline Characteristics

**Figure 1:**
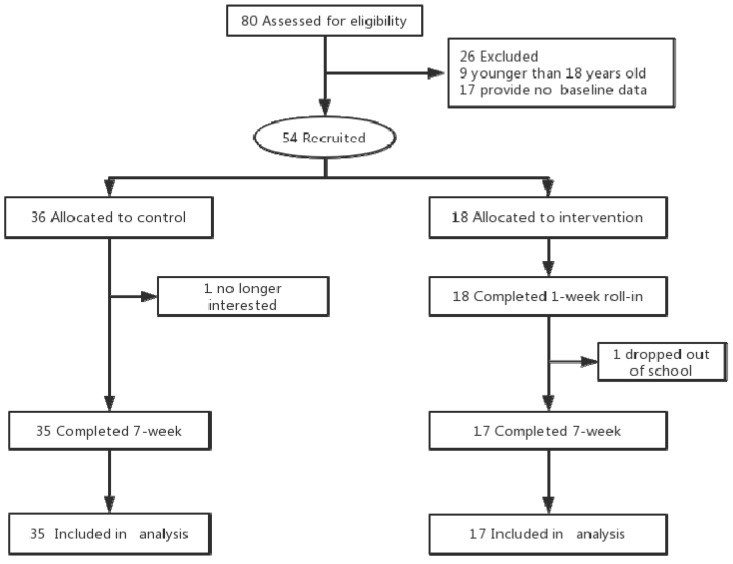
Study Flow Diagram.

Of the 54 participants recruited in this study, 18 were assigned to the intervention group, and 36 to the control group. However, 1 subject in the intervention group did not complete the study due to dropping out of school, and another subject in the control group dropped out due to lack of interest. A total of 52 subjects were included in the final data analysis (Figure 1).

The mean age was 20.76 (1.97) in the intervention group and 20.74 (2.33) in the control group, which were not significantly different (*p* = 0.958). A total of 9 subjects (52.94%) in the intervention group and 18 (51.43%) in the control group were female, which were not significantly different (*p* = 0.768). The mean BMI was not significantly different between the groups (*p* = 0.649), with a mean (SD) of 21.71 (2.50) and 21.36 (2.63) in the intervention group and control group, respectively. Physical activity measures, including baseline DPAD, DPAD increase from the baseline to the goal, vigorous physical activity (VPA) time, moderate physical activity (MPA) time, walking time, and sitting time were not significantly different between the groups (*p* = 0.760, 0.823, 0.549, 0.821, 0.050, 0.553, respectively). No TPB constructs showed significant between-group difference (*p* = 0.969, 0.346, 0.921, 0.129, respectively, for attitude, subjective norms, perceived behavior control, and intention). These indicated that the potential confounding factors were controlled, making the 2 groups comparable (see Table 2).

### 3.2. Intervention Effects on the Baseline (T0) to Post-Test (T1) Changes in the TPB Constructs.

The results of the independent sample Mann–Whitney U test (Table 3) showed that the means of attitude, subject norm, perceived behavior control, and intention for physical activity in the intervention group had increased from the baseline (T0) to the post-test (T1), while those TPB constructs had slightly waned in the control group, which showed significant differences between the two groups (*p* = 0.023, 0.006, 0.011, 0.000, respectively, for attitude, subjective norms, perceived behavior control, and intention). This indicated that the intervention led to a significant increase in TPB constructs for physical activity from the baseline (T0) to the post-test (T1) in the intervention group compared with the control group.

### 3.3. Intervention Effects on Baseline (T0) to Post-test (T1) Changes in Physical Activity Measures

The results of the independent sample Mann-Whitney U test (Table 4) demonstrated that the self-reported days doing VPA per week, VPA time per day on days doing VPA, and total VPA time per week, as well as the days doing MPA per week, MPA time per day on days doing MPA, and total MPA time per week in the intervention group had increased after the intervention, and the changes were significantly different from those in the control group (*p* = 0.000, 0.019, 0.000, 0.012, 0.013, 0.000, respectively). This indicated that the intervention effectively promoted VPA and MPA in the intervention group compared with the control group. Accumulated sitting time per week had decreased in the intervention group from the baseline (T0) to the post-test (T1), with a mean (SD) change of −420.00 (410.41) min in the intervention group and 0.20 (544.05) min change in the control group, which showed significant difference between the two groups (*p* = 0.005). However, changes in the days walking >10 min per week, walking time per day on those days, and accumulated walk time per week did not show significant differences between the two groups, with a slight increase found in both groups. Overall, the total score of physical activity measured by IPAQ had increased in both groups after the intervention, with a mean (SD) change of 1497.12 (640.62) min in the intervention group and 361.86 (974.64) min change in the control group, yet the increase in the intervention group was significantly greater than the control group (*p* = 0.000).

**Figure 2:**
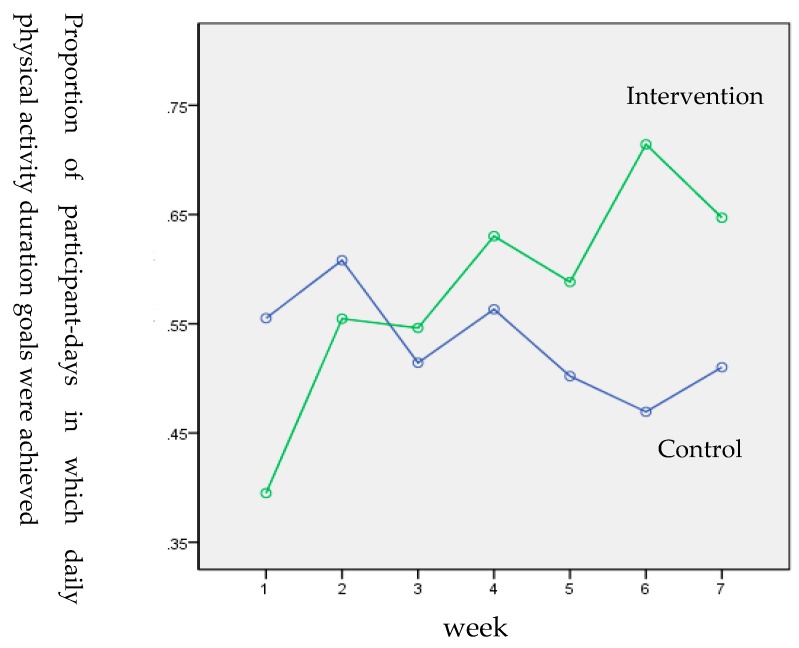
Proportion of participant-days in which daily physical activity duration goals were achieved by group and week.

The results of the ANOVA with repeated measurements showed an interaction effect of group×time for the proportion of participant-days in which the DPAD goals were achieved per week (adjusted F _4.102, 205.112_ = 4.206, *p* = 0.002, η^2^_partial_ = 0.10) with large effect size. During the 7 weeks from the baseline (T0) to the post-test (T1), the proportion of participant-days in which the DPAD goals were achieved per week in the intervention group showed a general ascending trend, while it declined to a small extent in the control group (see Figure 2).

**Figure 3:**
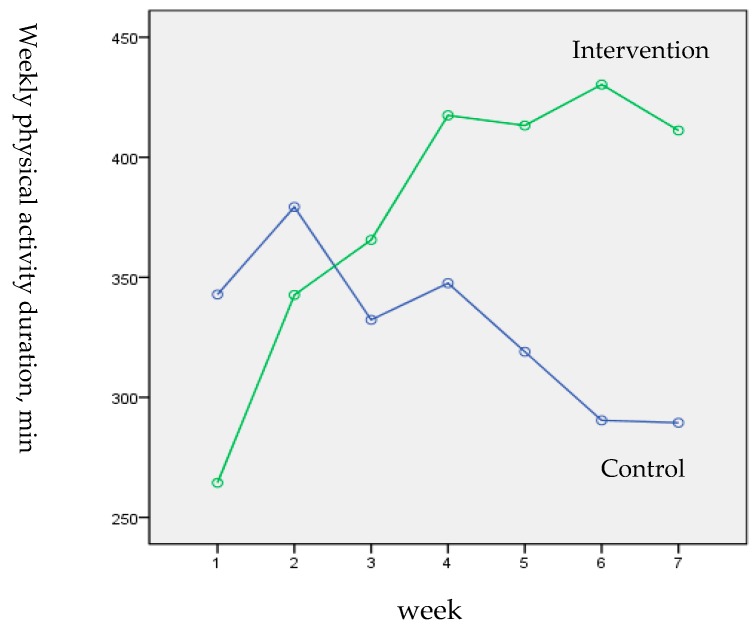
Daily physical activity duration by study group and week.

The results of the ANOVA with repeated measurements showed an interaction effect of group×time for accumulative weekly physical activity duration (adjusted F _4.625, 231.245_ = 6.849, *p* = 0.000, η^2^_partial_ = 0.12) with large effect size. During the 7 weeks from the baseline (T0) to the post-test (T1), the accumulative weekly physical activity duration in the intervention group showed a general ascending trend, while it declined to a small extent in the control group (see Figure 3).

### 3.4. Prediction of PA by TPB Constructs

**Figure 4:**
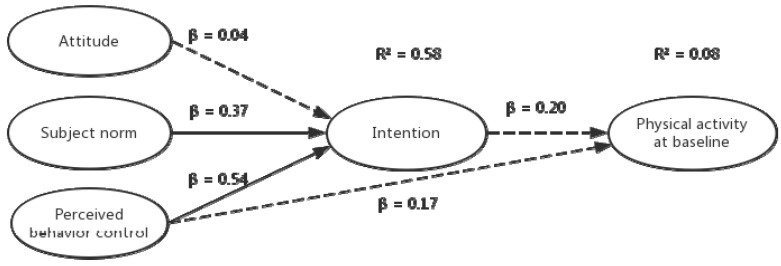
Path diagram for the TPB constructs assessed at baseline predicting PA.

Figure 4 demonstrates the path diagram for the TPB constructs assessed at the baseline (T0), predicting PA total score measured by IPAQ. In this model, the subjective norms (β = 0.37; *p* <0.01) and perceived behavioral control (β = 0.54; *p* <0.01) independently predicted intention and explained 58% of its variance. No TPB construct predicted PA total score during this period (*p* >0.05), including a second block in the regression analyses where all the TPB constructs were freed as paths to predict self-reported behavior.

**Figure 5:**
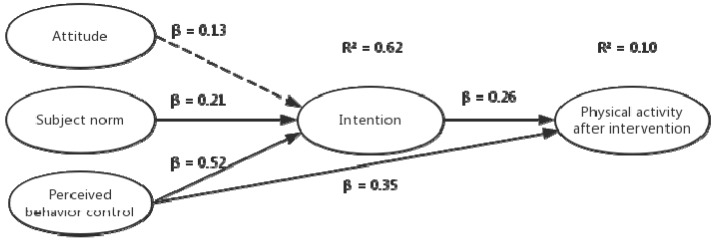
Path diagram for the TPB constructs assessed after intervention predicting PA.

Figure 5 shows the path diagram for the TPB constructs assessed after intervention, predicting PA total score. In agreement with the model at the baseline, the subjective norms (β = 0.21; *p* <0.05) and perceived behavioral control (β = 0.52; *p* <0.05) independently predicted intention and explained 62% of its variance. Moreover, perceived behavior control (β = 0.35; *p* <0.05) and intention (β = 0.26; *p* <0.05) independently predicted PA total score and explained 10% of its variance.

## 4. Discussion

The main purpose of this study was to evaluate whether a WeChat-based intervention could improve subjectively measured PA, as well as related cognitive constructs. We hypothesized that subjectively measured PA and related TPB constructs of the intervention group would increase after the intervention compared with the control group, and the results supported this hypothesis.

Attitude, subjective norms, perceived behavior control, and intention toward PA in the intervention group showed increases after the intervention, yet the control group showed slight waning trends, confirming the intervention effect on the PA-related cognitive variables. This was in accordance with previous interventions targeting social cognitions about PA [25] and also corresponded with the intervention design. Sending educational materials about PA aimed to make subjects aware of the benefit of enough PA and the hazard of physical inactivity, thereby attempting to change their attitudes toward PA. The different trends in the two study groups also indicated that mere education, without making the subjects fully understand and identify the knowledge, might not cause a change in attitude, let alone PA behavior. That is also the reason we integrated many practical designs of gamification and social incentives into the intervention. The social incentives applied in this intervention, such as peer support (accompaniment, praise, encouragement, and help), competition, and discussion, enhanced the influence that the subjects could receive from their peers, which resulted in the increase of the subjective norms in the intervention group. Working in teams rather than individually made the subjects feel responsible for their teammates and therefore encouraged them try to meet the requirements to achieve the shared objective and honor their teams. Working with peers was easier than struggling alone. With the praise, encouragement, and help from their teammates, their perceived behavior control about PA also improved. To stimulate enduring engagement in the intervention, it was necessary to make the subjects realize that maintaining enough PA was not only beneficial, but also interesting, and gamification features were integrated to make the process enjoyable. The gamified designs in this intervention (points, ranking, punishments, and rewards) seemed to leverage positive emotions, such as interest, enthusiasm, inspiration, and attention to promote cognitive engagement, as a previous study inferred [33], because the subjects showed passion for their points and ranking. When a team was punished to perform a talent show, the atmosphere in the WeChat group became much more active and delightful. Using lively expressions and friendly jokes, the number of messages in the group soared up when people tried to urge the punished team to perform before the show and commented on the show afterwards. “The talent show absolutely made my day” said one student “It is the most enjoyable thing in the whole week for me.”

More importantly, this intervention demonstrated significant effects on increasing VPA and MPA. Consistent with some of previous studies [13,49], these results indicated that this intervention promoted subjectively measured PA. The average increase of more than 100 minutes of VPA and MPA per week exceeded 66% of the recommended amount by the WHO [5], which indicated notable health benefits and appreciably reduced the risk of many noncommunicable disease [1,2,3,4,50]. This extent of increase is also significant for decreasing the burden of disease and mortality, as well as increasing life expectancy [1,3,4]. As a cogent study estimated previously, if physical inactivity decreased by 10% or 25%, more than 533,000 and more than 1.3 million deaths worldwide, respectively, could be averted every year, and the elimination of physical inactivity would increase the life expectancy of the world’s population by 0.68 (range 0.41–0.95) years [3]. Furthermore, from the recorded DPAD in the 7-week intervention, we could see the generally upward trend of the intervention group, which indicated that the intervention could exert continuous motivation for the subjects to achieve their DPAD goals. The downtrend of DPAD in the control group also pointed out that even in those who had relatively strong intentions to keep fit, if they could not receive effective guidance and continuous motivation, their enthusiasm would wane with time naturally, as striving to increase PA is not always interesting itself. Another noteworthy change was that the sitting time in the intervention group decreased by around 7 hours per week. As previous studies emphasized, even when adults meet PA guidelines, sitting for prolonged periods can still compromise metabolic health [51]. Interestingly, this intervention did not show effectiveness with respect to walking. This might be because the participants were energetic youth who may have interpreted PA strictly as VPA or MPA and did not consider walking as a viable PA format. It is worth noting that PA behavior measured by self-reported scales might not accurately reflect actual amounts, but it was a reasonable estimate and could be used to compare the PA levels in the two groups. This is because previous studies had provided evidence that the agreement between subjective and objective methods for assessing PA intensity and duration was moderate [52], with higher agreement found for younger age groups (18–34 years) [53]. Furthermore, sample sizes ranging from 50 to 99 subjects can provide stable agreement estimates between the two methods [52]. Thus, the increase of PA in the intervention group, compared with the control group, should be considered as credible.

Consistent with previous research [21,49], this study showed that TPB constructs were correlated with perceived PA behavior, and intention was the strongest determinant. In the model in this study, subjective norms and perceived behavior control showed significant prediction of intention. Attention needs to be paid to subjective norms, as most of the designs in this intervention mainly aimed to improve this construct. In previous research, subjective norms have sometimes been the least powerful predictor among the TPB variables [13]. However, when it comes to the younger generation, subjective norms become more important, because their behavior is influenced by their social contact to a large extent [25,54]. The design of this intervention was effective because it fits the characteristics of undergraduates. Because undergraduates usually feel good about their health and rarely notice the threat of diseases, educating them about the benefits of PA and the threat of physical inactivity might not correspond to their personal demands. This also might be the reason many previous interventions focusing on education had little effect [21,55]. Rather than to improve health, the main purposes for undergraduates to exercise are to obtain an attractive figure, to pursue the keeping fit trend so that they look fashionable, to make more friends, or just to have a better mood. Obviously, undergraduates, compared to people at other ages, have more mental and emotional demands towards exercise. These demands need to be positively reinforced by people around them to become a motivating force. This explains why our intervention, which relied on the influence of peers in social networks, was found to be effective.

This study has practical implications. It demonstrates how to identify gamification and social incentives features suitable for undergraduate students and how to integrate these features into a web-based behavior intervention. It sets an exemplar case by showing the effectiveness of such an intervention, which can inform the design and implementation of future PA interventions for undergraduate students.

### Strengths and Limitations

We creatively designed a WeChat-based intervention that integrated gamification and social incentives to increase PA and conducted a controlled trial to demonstrate its effectiveness on the promotion of PA behavior, as well as related TPB constructs. This type of intervention implemented through social networking services provided an easy, effective, and inexpensive mode for improving undergraduates’ compliance and commitment, as well as passion for increasing PA, thus making it possible to maintain long-term behavior change. The researchers did not need to expend much effort in the intervention, as it helped the participants develop intrinsic motivation, and the mechanism could be run by the participants themselves. So, the scope of this study not only applied to the intervention period, but also aimed at helping to form a healthy habit to obtain lifelong benefits. Moreover, this study also gave light to the application of WeChat in behavior change programs, offering a reference for making adequate use of this leading, popular, and free instrument.

On the other hand, this study had several limitations. First, the data of PA behavior were measured through self-reported subjective measures, and the accuracy remains uncertain. However, as a relatively convenient, cheap, and operable approach to measuring PA, self-reported scales have been widely utilized in many studies and have obtained satisfactory reliability in reflecting accurate PA behavior [15,20,56]. The past-day recall of DPAD has shown applicability in health behavior surveillance studies [57,58], and the past-day recall questionnaire has provided acceptable agreement with PA time measured by device [58]. The single-item measure of DPAD used in this study was found to perform as well as other short physical activity tools in terms of reliability and validity [55,59]. Nevertheless, it is possible that the subjects tended to increase their self-reported DAPD over time. Future research could utilize objective measures to more definitely evaluate the intervention effect. Second, the intervention period in this study was relatively short and did not include a follow-up. Thus, the long-term effect of the intervention remains unclear. However, there is evidence that even a 6-week intervention could obtain significant changes in TPB constructs and PA behavior [39,42]. A recent longitudinal study confirmed that a 6-week PA intervention was enough to improve human gut microbiota, independent of diet, which indicated that even 6 weeks of increase in PA could induce considerable health benefits [60]. Future research needs to include follow-up observations to study whether the positive effects can be sustained over time. Third, the subjects were not completely independent, because the subjects in each condition were able to interact, and the intervention design required social interaction within the teams. More granular data should be collected to address this problem. Last but not least, because we integrated designs of gamification and social incentives into the intervention, the effect of gamification and social incentives could not be disentangled. Future research could include separate intervention groups with/without gamification and with/without social incentives to identify the distinct effect of gamification and social incentives.

## 5. Conclusions

In conclusion, the WeChat-based intervention integrating features of gamification and social incentives in this study was effective at increasing self-reported physical activity, as well as related TPB constructs among undergraduates in China. Our findings suggest that gamification and social incentives may offer a promising approach to promoting health behavior change in young people. 

## Figures and Tables

**Table 1:** Recommended standard for setting the daily physical activity duration (DPAD) goals for the subjects in this study.

Baseline Weekly PA Duration (min)	Percentage of Goal Greater than the Baseline
150~	0~10%
120~149	10~20%
90~119	20~30%
60~79	30~40%
30~59	40~60%
~30	60~80%

**Table 2:** Baseline characteristics of the study participants.

Variable	Intervention Group (*n* = 17)	Control Group (*n* = 35)	*p*
**Demographics**			
Age, mean (SD), y	20.76 (1.97)	20.74 (2.33)	0.958
Female, No. (%)	9 (52.94)	18 (51.43)	0.768
Baseline Measures			
BMI, mean (SD), kg/m^2^	21.71 (2.50)	21.36 (2.63)	0.649
**Physical Activity Measures**			
DPAD, mean (SD), min	36.39 (27.26)	48.98 (22.10)	0.760
DPAD increase from the baseline to the goal, mean (SD), min	25.86 (11.37)	30.59 (14.29)	0.823
days doing VPA per week, mean (SD), d	2.24 (1.35)	2.94 (1.63)	0.118
VPA time per day, mean (SD), min	26.18 (14.42)	26.57 (15.38)	0.784
VPA time per week, mean (SD), min	72.65 (50.93)	89.71 (64.99)	0.549
days doing MPA per week, mean (SD), d	2.88 (1.45)	3.03 (2.15)	0.890
MPA time per day, mean (SD), min	31.47 (18.61)	31.71 (19.78)	0.676
MPA time per week, mean (SD), min	87.35 (50.50)	113.71 (105.04)	0.821
Days Walking>10min per week, mean (SD), d	6.53 (0.72)	5.94 (1.21)	0.113
Walk time per day, mean (SD), min	47.94 (28.23)	43.14 (37.26)	0.192
Walk time per week, mean (SD), min	306.18 (181.52)	267.71 (259.72)	0.050
Sitting time per week, mean (SD), min	3306.47 (590.62)	3455.80 (870.82)	0.553
Physical activity total score, mean (SD)	1940.97 (813.28)	2056.03(1323.26)	0.585
**TPB Construct Measures**			
Attitude, mean (SD)	5.51 (1.28)	5.53 (0.99)	0.969
Subjective norms, mean (SD)	5.27 (1.09)	5.58 (1.02)	0.346
Perceived behavioral control, mean (SD)	5.97 (0.96)	5.98 (1.06)	0.921
Intention, mean (SD)	5.65 (0.89)	5.99 (1.19)	0.129

BMI = body mass index (calculated as weight in kilograms divided by height in meters squared); DPAD = daily physical activity duration; VPA = vigorous physical activity; MPA = moderate physical activity; TPB = theory of planned behavior

**Table 3:** Changes in the TPB constructs from the baseline (T0) to the post-test (T1).

Variable	Intervention group (N = 17)	Control group (N =3 5)	*P*
Attitude, mean (SD)	0.55 (1.11)	−0.13 (1.08)	0.023
Subjective norms, mean (SD)	0.82 (1.13)	−0.17 (1.23)	0.006
Perceived behavioral control, mean (SD)	0.28 (0.90)	−0.35 (0.97)	0.011
Intention, mean (SD)	0.80 (0.83)	−0.40 (1.23)	0.000

**Table 4:** Changes in physical activity measured from the baseline (T0) to the post-test (T1).

Variable	Intervention Group (*n* = 17)	Control Group (*n* = 35)	*p*
days doing VPA per week, mean (SD), d	2.29 (1.11)	0.66 (1.80)	0.000
VPA time per day, mean (SD), min	12.94 (13.92)	2.71 (12.14)	0.019
VPA time per week, mean (SD), min	105.59 (77.43)	18.14 (56.10)	0.000
days doing MPA per week, mean (SD), d	1.41 (1.27)	0.09 (1.82)	0.012
MPA time per day, mean (SD), min	13.24 (22.63)	−2.14 (16.51)	0.013
MPA time per week, mean (SD), min	101.47 (75.74)	−3.57 (72.28)	0.000
Days Walking>10 min per week, mean (SD), d	0.47 (0.71)	0.91 (1.22)	0.256
Walk time per day, mean (SD), min	6.47 (20.37)	7.00 (34.30)	0.735
Walk time per week, mean (SD), min	74.71 (124.09)	70.00 (225.87)	0.937
Sitting time per week, mean (SD), min	−420.00 (410.41)	0.20 (544.05)	0.005
Physical activity total score, mean (SD)	1497.12 (640.62)	361.86 (974.64)	0.000

VPA = vigorous physical activity; MPA = moderate physical activity.
